# Lysosomes contribute to the synthesis of tricarboxylic acid-related metabolites in the hippocampus

**DOI:** 10.1093/lifemeta/loag005

**Published:** 2026-02-19

**Authors:** Liangliang Kong, Hongying Tan, Xiaoting Zhu, Yiqing Wen, Yang Li

**Affiliations:** Department of Pharmacology, State Key Laboratory of Brain Function and Disorders and MOE Frontiers Center for Brain Science, Key Laboratory of Metabolism and Molecular Medicine, Ministry of Education, School of Basic Medical Sciences, Fudan University, Shanghai 200032, China; Department of Pharmacology, State Key Laboratory of Brain Function and Disorders and MOE Frontiers Center for Brain Science, Key Laboratory of Metabolism and Molecular Medicine, Ministry of Education, School of Basic Medical Sciences, Fudan University, Shanghai 200032, China; Department of Pharmacology, State Key Laboratory of Brain Function and Disorders and MOE Frontiers Center for Brain Science, Key Laboratory of Metabolism and Molecular Medicine, Ministry of Education, School of Basic Medical Sciences, Fudan University, Shanghai 200032, China; Department of Pharmacology, State Key Laboratory of Brain Function and Disorders and MOE Frontiers Center for Brain Science, Key Laboratory of Metabolism and Molecular Medicine, Ministry of Education, School of Basic Medical Sciences, Fudan University, Shanghai 200032, China; Department of Pharmacology, State Key Laboratory of Brain Function and Disorders and MOE Frontiers Center for Brain Science, Key Laboratory of Metabolism and Molecular Medicine, Ministry of Education, School of Basic Medical Sciences, Fudan University, Shanghai 200032, China

**Keywords:** lysosome, Lyso-IP, TCA cycle, metabolomics, mouse hippocampus

## Abstract

The central nervous system is highly sensitive to energy supply, and the hippocampus operates under sustained metabolic load due to continuous synaptic activity and information processing. Lysosomes couple nutrient status to cellular energetics through the mechanistic target of rapamycin complex 1 (mTORC1) and the autophagy–lysosome pathway, yet their ­subcellular contribution to neuronal metabolic profiles remains unclear. To address this, we established an *in vivo* AAV-LysoTag/Lyso-IP workflow combined with metabolomics to quantify metabolites within mouse hippocampal lysosomes. An *in vitro* Lyso-IP platform and immunofluorescence provided cell-based validation. Under every-other-day fasting, hippocampal lysosomes exhibited reprogramming: small-molecule substrates derived from amino acids and fatty acids accumulated; bis(monoacylglycero)phosphate was upregulated, indicating enhanced intraluminal vesicle formation and lipid degradation/sorting; ­sphingolipids and cardiolipin increased, consistent with selective mitophagy. Notably, high basal lysosomal levels of malic acid and α-ketoglutarate (α-KG) suggested additional sources beyond the mitochondria. Immunofluorescence further showed lysosomal localization of isocitrate dehydrogenase and fumarate hydratase, suggesting partial residency of these enzymes. The oxoglutarate carrier (SLC25A11) signals were observed in LAMP1^+^ compartments, suggesting potential transmembrane exchange of α-KG and malic acid. Together, our data indicate that lysosomal tricarboxylic acid -related metabolites are maintained by three parallel routes: mitochondrial delivery to lysosomes, local production by resident enzymes, and transporter-mediated exchange. These metabolites supplement and reshape neuronal carbon flux and metabolic resilience at the subcellular level. Our findings elevate lysosomes from degradative endpoints to mobilizable metabolic hubs in the brain and provide both methodological and conceptual frameworks for neurometabolic adaptation under energy scarcity.

## Introduction

Eukaryotic cells compartmentalize metabolism across distinct organelles, each executing specialized functions that cooperate to support growth and homeostasis [1]. Traditionally, lysosomes have been viewed as cellular “incinerators”: within an acidic lumen maintained by V-ATPase, they house more than 60 acid hydrolases that degrade proteins, lipids, polysaccharides, and nucleic acids into amino acids, nucleosides, and sugars [1–3]. Recent work shows, however, that these catabolites do not remain in the lysosomal lumen; instead, they are exported to the cytosol via specific lysosomal membrane transporters and reutilized for anabolic biosynthesis or catabolic energy production [3]. Importantly, lysosomes have emerged as integrative hubs for nutrient sensing and metabolic signaling: the mechanistic target of rapamycin complex 1 (mTORC1) and related complexes are organized on the lysosomal membrane, and lysosome-derived signaling pathways regulate nutrient sensing, metabolic adaptation, inter-organelle communication, and even aging [2–4]. Consequently, lysosomes are no longer considered as mere degradative endpoints, but as centers of metabolic integration that are essential for cellular homeostasis. Nevertheless, a comprehensive *in vivo* atlas of ­lysosomal metabolites and lipids is still lacking. Recent LysoTag/lysosomal immunoprecipitation (Lyso-IP) advances allow rapid, selective purification of intact lysosomes for multi-omics and furnish the methodological basis to fill this gap [5, 6].

Our previous work has shown that enhancing lysosomal ­biogenesis/function facilitates the clearance of toxic proteins in the hippocampus and other brain regions, and improves Alzheimer’s disease–related phenotypes, highlighting a critical role for hippocampal lysosomal homeostasis in neurodegenerative patho­logy [7–9]. Moreover, from a physiological perspective, the brain is a metabolically active organ with high energy demand and rapid substrate turnover [10–14]. Hippocampal neurons, which are essential for synaptic plasticity and memory formation, are particularly sensitive to fluctuations in nutrient availability, and the autophagy–lysosome pathway represents a central hub for maintaining intracellular proteostatic and metabolic homeostasis [15–17]. Therefore, we focused on the hippocampus to systematically delineate the dynamic remodeling of the neuronal lysosomal metabolome under distinct nutritional states and to provide testable directions and candidate targets for subsequent mechanistic studies. Notably, the hippocampus also offers well-established stereotaxic injections and region-specific sampling strategies, which further enhance the feasibility and reproducibility of our *in vivo* analyses. Under nutrient deprivation, cells undergo metabo­lic reprogramming to maintain energy and substrate homeostasis, primarily by activating the autophagy–lysosome pathway to deliver cytosolic components for degradation [18–20]. Lysosomes are the central effectors of these responses: when nutrients are abundant, active mTORC1 inhibits the assembly of the V-ATPase, thereby limiting lysosomal acidification; conversely, during starvation, mTORC1 becomes inactive, V-ATPase reassembles, luminal pH decreases, and proteolytic capacity increases [21]. In parallel, starvation relieves inhibition of transcription factor EB (TFEB), promoting its nuclear translocation and induction of lysosomal/autophagy gene programs that support metabolic adaptation [9]. Beyond degradation, lysosomes regulate metabo­lite flux through dedicated membrane transporters: for example, SLC38A9 senses/exports arginine to coordinate the mTORC1 signaling [3], whereas cationic amino acid transporters such as PQ-loop repeat-containing protein 2 (PQLC2) (and lysosomal amino acid transporter 1 homologue (LAAT-1) in *Caenorhabditis elegans*) mediate lysosomal amino acid efflux to sustain intracellular pools [22–24]. Collectively, these mechanisms position lysosomes as dynamic hubs that integrate nutrient sensing, acidification control, and metabolite trafficking with system-level metabolic reprogramming. However, in the brain under nutrient deprivation, there is currently no *in vivo* atlas of lysosomal metabolites and lipids.

The tricarboxylic acid (TCA) cycle is a central metabolic pathway that oxidizes the breakdown products of carbohydrates, fats, and amino acids in the mitochondrial matrix, generating energy mole­cules such as ATP and NADH [25, 26]. Beyond bioenergetics, TCA intermediates supply precursors for biosynthesis and enact regulatory signaling [26]. For example, citrate supports fatty acid and cholesterol synthesis; α-ketoglutarate (α-KG) serves as a cofactor for transamination and α-KG–dependent dioxygenases involved in epigenetic regulation; succinate inhibits prolyl hydroxylases to stabilize the transcription factor hypoxia-inducible factor-1alpha (HIF-1α); and fumarate and malic acid participate in redox and immune control [27]. These observations indicate that TCA metabolites function broadly outside canonical mitochondrial energy production. Although the TCA cycle is classically mitochondrial, several enzymes reside in additional compartments. Isocitrate dehydrogenase-1 (IDH1) localizes to the cytosol and peroxisomes [27], malate dehydrogenase-1 (MDH1) operates in the cytosol as part of the malate–aspartate shuttle with mitochondrial MDH2 [28], and fumarase (fumarate hydratase, FH) is detected in the cytosol and nucleus and contributes to the DNA damage response [29]. Recent advances in lysosome isolation and metabolomics have revealed small-molecule metabolites, including amino acids and organic acids, within the lysosomal lumen, underscoring the role of the lysosome in metabolite handling and egress [5]. In *Drosophila*, starvation-induced mobilization of lysosomal ­cystine can feed acetyl coenzyme A (acetyl-CoA) production, elevate cellular TCA intermediates, and restrain the growth regulator target of rapamycin complex 1 (TORC1) reactivation, linking lysosomal outputs to central carbon metabolism [30]. Nevertheless, direct evidence that lysosomes autonomously execute substantial segments of the TCA cycle remains limited. The physiological significance of lysosome-localized TCA intermediates—whether as transient stores, transported substrates, or local signals—requires further elucidation.

In this study, we established an *in vivo* adeno-associated virus (AAV)-LysoTag/Lyso-IP workflow in the mouse hippocampus and combined it with metabolomics to analyze the remodeling of lyso­somal metabolite and lipid profiles under every-other-day fasting (EODF); we then further focused on TCA-related metabolism. We systematically quantified EODF-driven enrichment of amino acid– and fatty acid–derived small molecules within lysosomes, together with changes in lipids, such as bis(monoacylglycero)phosphate (BMP), sphingolipids, and cardiolipin (CL), which indicate membrane turnover and autophagy activity. Drawing on Lyso-IP and imaging evidence, we delineated the *in vivo* distribution and putative sources of lysosomal TCA intermediates and key enzymes. We propose that these lysosomal TCA intermediates are contributed by three parallel pathways: (i) mitochondrial delivery to lysosomes; (ii) local generation by resident enzymes; and (iii) transporter-mediated exchange. Using this methodological framework, we aimed to define how hippocampal lysosomes couple to core carbon-metabolic networks, to explain the lysosomal metabolic switch under energy scarcity, and to provide clear experimental avenues for subsequent mechanistic studies on lysosomal roles in energy homeostasis and neurodegenerative pathology.

## Results

### Lyso-IP enables rapid immunoisolation of intact lysosomes for absolute quantification of intralysosomal metabolites

**Figure 1:**
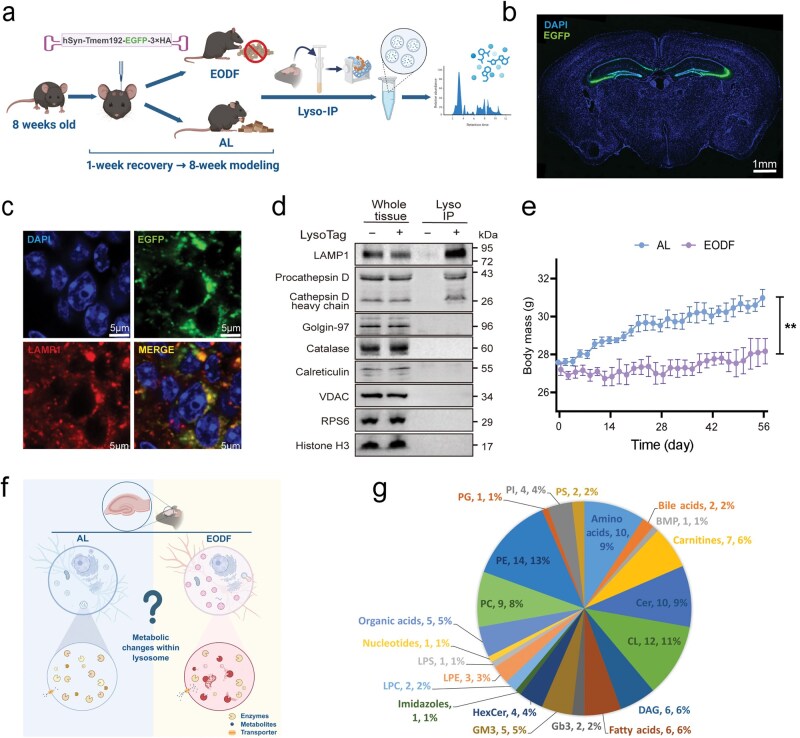
Lyso-IP enables rapid immunoisolation of intact lysosomes for absolute quantification of intralysosomal metabolites. (a) Schematic diagram of the animal model and Lyso-IP workflow. EODF, every-other-day fasting; AL, *ad libitum* feeding. (b) EGFP expression in the hippocampus. Imaging was performed 4 weeks after AAV injection. Scale bar, 1 mm. The image is representative of three independent experiments. (c) TMEM192-EGFP-3×HA tag (EGFP) and immunofluorescence labeling of lysosomes with anti-LAMP1 in the hippocampus. Nuclei were stained with DAPI. Imaging was performed 4 weeks after AAV injection. Scale bar, 5 µm. The image is representative of three independent experiments. (d) Immunoblot analysis of subcellular markers in whole hippocampal tissue, purified lysosomes (IP LysoTag^+^), and control immunoprecipitates (IP LysoTag^–^). Golgin-97, catalase, calreticulin, VDAC, RPS6, and histone H3 mark the Golgi apparatus, peroxisome, endoplasmic reticulum, mitochondria, ribosome, and the nucleus, respectively; LAMP1 and cathepsin D mark the lysosomal membrane and lumen, respectively. Blots are representative of three independent experiments. (e) Body weight trajectories of AL and EODF mice. *n *= 4 for AL group; *n *= 3 for EODF group. (f) Strategy to identify differentially abundant metabolites within hippocampal lysosomes of EODF mice. Created with BioRender.com. (g) Metabolomics of Lyso-IP isolates from mouse hippocampus. Pie chart of lysosomal metabolite species identified by comparison with control IP (LysoTag^−^) metabolomes. *n *= 4 for ALgroup; *n *= 3 for EODF group. For all quantifications, data (mean ± SEM) are from the indicated number of independent experiments and analyzed with the two-tailed unpaired *t*-test. ^**^P < 0.01.

To precisely quantify intralysosomal metabolites under nutrient deprivation (induced by EODF), we established a workflow involving stereotaxic delivery to the hippocampus of AAV expressing the lysosome-specific protein TMEM192 (TMEM192-EGFP-3×HA), followed by purification of lysosomes via Lyso-IP using anti-HA magnetic beads (Fig. 1a). Four weeks after injection of the AAV into the mouse brain, microscopic observation revealed strong EGFP fluorescence in the hippocampal region. This result indicates that the TMEM192-EGFP-3×HA fusion protein is actively expressed in the hippocampus (Fig. 1b). Furthermore, we confirmed the precise localization of TMEM192-EGFP-3×HA within lysosomes by observing extensive overlap between EGFP fluorescence and immunofluorescence for another lysosome-specific protein, lysosome-associated membrane protein 1 (LAMP1), in hippocampal slices (Fig. 1c). Western blotting was used to verify the purity of the Lyso-IP fraction (Fig. 1d). The lysosomal membrane marker LAMP1 was markedly enriched. Consistent with the specific enrichment of LAMP1, we detected a strong signal for the mature heavy chain of cathepsin D (CTSD). Notably, we also observed bands corresponding to pro-CTSD. The presence of this immature form within the purified lysosomal fraction is physiologically consistent with the stepwise maturation process of CTSD in the lysosomal system [31–33]. CTSD trafficking involves the delivery of the inactive pro-form to endosomes/lysosomes, where it undergoes sequential proteolytic cleavage into an intermediate form and ultimately the mature form [31–33]. Since the TMEM192-based Lyso-IP method isolates intact organelles, including those in dynamic states of fusion and maturation (e.g. endo-lysosomes), the presence of these intermediate forms reflects the active flux of newly synthesized lysosomal hydrolases being delivered to ly­sosomes. In contrast, non-lysosomal markers, Golgin-97 (Golgi), catalase (peroxisome), calreticulin (endoplasmic reticulum), voltage-dependent anion channel (VDAC for mitochondrium), ­ribosomal protein S6 (RPS6 for ribosome), and histone H3 ­(nucleus), were nearly undetectable, indicating high purity with minimal contamination from other organelles. Body weight monitoring revealed a clear decrease in EODF mice relative to *ad libitum* (AL)-fed controls (Fig. 1e), reflecting the physiological impact of intermittent fasting. To investigate metabolic changes in lysosomes under nutritional deprivation, we performed metabolomics analysis on purified hippocampal lysosomes to determine EODF-induced alterations in lysosomal metabolite levels (Fig. 1f). After background correction using a control immunoprecipitate (LysoTag^−^), we identified a total of 109 metabolites in purified lysosomes from mouse hippocampus, including lipids, amino acids, organic acids, carnitine, and other metabolites.

In summary, we have established an AAV-mediated lysosomal isolation platform, laying a solid foundation for subsequent lysosome-centered metabolomics analysis under nutrient-deprived conditions.

### Elevated TCA cycle metabolites in the EODF group

**Figure 2:**
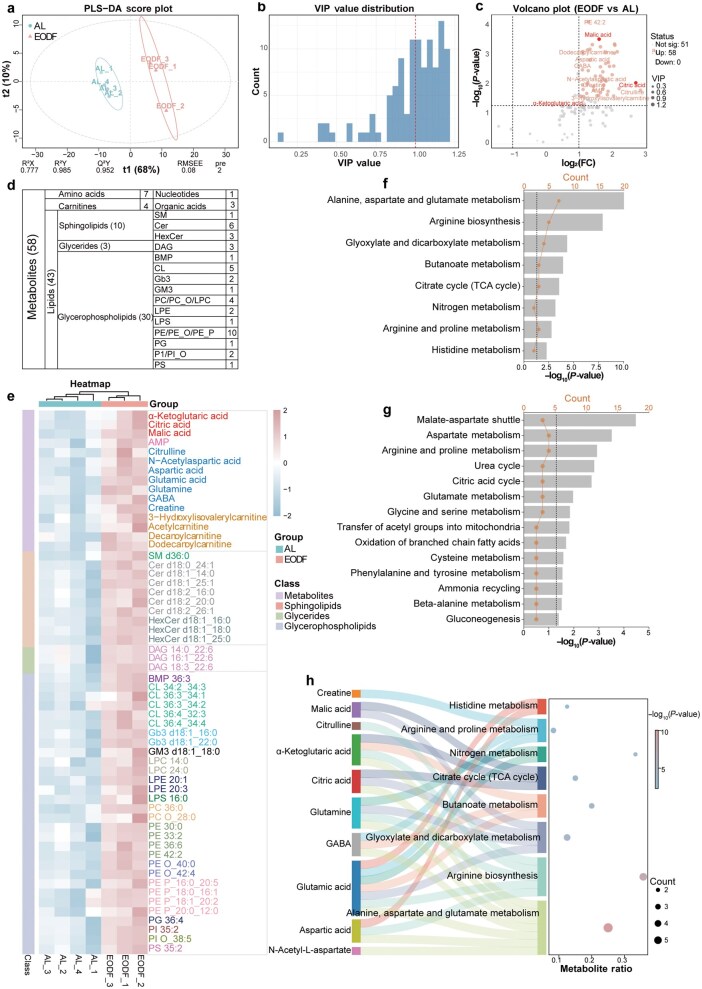
Metabolomic analysis showing that organic metabolites associated with the TCA cycle are significantly up-regulated in the EODF group. (a) PLS-DA score plot for the EODF and AL groups. (b) Bar chart of VIP value distribution with thresholds (dashed lines). (c) Volcano plot showing 58 metabolites that are significantly up-regulated in the EODF group. Key TCA cycle metabolites are highlighted. (d) Grouping of the 58 differential metabolites into different chemical classes. (e) Heatmap showing the levels of the 58 differential metabolites in EODF and AL samples. (f and g) KEGG (f) and SMPDB (g) enrichment pathway analyses of the 58 differential metabolites. (h) Sankey diagram based on the enriched KEGG pathways and their associated metabolites. *n *= 4 for AL group; *n *= 3 for EODF group.

To systematically characterize the metabolic alterations induced by EODF, we performed targeted metabolomics and lipidomics profiling of hippocampus tissues. The partial least squares discriminant analysis (PLS-DA) metabolite model (Fig. 2a) achieved distinct separation between EODF and AL groups (R^2^Y = 0.985), with high explanatory power for metabolic profiles (R^2^X = 0.777) and robust predictive accuracy (*Q*^2^ = 0.952, 7-fold cross-validation). The low estimation error (RMSEE = 0.08) and small predictive residuals (PRE = 2) further supported model reliability. In addition, the clear separation between EODF and AL groups in the PLS-DA model (Fig. 2a) was driven by a subset of metabolites with high contribution weights. Variable importance in projection (VIP) analysis identified 61 metabolites with VIP scores ≥ 1.0 (Fig. 2b). A volcano plot, combining multivariate analysis (VIP > 0.9) with univariate criteria (*P *< 0.05 and |log_2_(FC)| > 1) as metabolite screening thresholds (Fig. 2c), revealed that 58 out of the 109 altered metabolites in Fig. 1g were significantly upregulated in the EODF group. These 58 metabolites were composed of 43 lipids (10 sphingolipids, 3 glycerol esters, and 30 glycerophospholipids), 7 amino acids, 4 carnitines, 3 organic acids, and 1 nucleotide (Fig. 2d). To visualize the levels of these 58 differentially abundant metabolites across samples, we performed within-group clustering analysis. As shown in Fig. 2e, the heatmap demonstrates a striking dichotomy between groups, with all AL samples exhibiting uniformly low expression (blue) and EODF samples showing consistent upregulation (pink) across all 58 metabolites. Subsequently, to decipher the biological implications of these 58 metabolites, we combined Kyoto Encyclopedia of Genes and Genomes (KEGG) and SMPDB databases for pathway enrichment analysis. A total of 10 differential metabolites matched in the KEGG database were significantly enriched in eight pathways, including amino acid metabolism (alanine, aspartate, and glutamate metabolism, arginine biosynthesis, arginine and proline metabolism, and histidine metabolism), carbohydrate metabolism (glyoxylate and dicarboxylate metabolism, butanoate metabolism, and citrate cycle), and energy metabolism (nitrogen metabolism) (Fig. 2f and h). In addition, we identified 14 SMPDB pathways that exhibited significant enrichment, with the malate–aspartate shuttle pathway (which maintains NAD^+^/NADH equilibrium) displaying both the broadest metabolite coverage and the greatest statistical significance (as ­reflected by the lowest *P*-value) (Fig. 2g). The integration of pathway enrichment (Fig. 2f and g) and flux analysis (Fig. 2h) reveals that EODF preferentially remodels three key TCA cycle intermediates, malic acid, α-KG, and citric acid, which serve as pivotal hubs linking carbon and nitrogen metabolism (alanine/aspartate/glutamate metabolism).

### System-wide metabolic network reconstruction reveals EODF-induced pathway crosstalk

**Figure 3:**
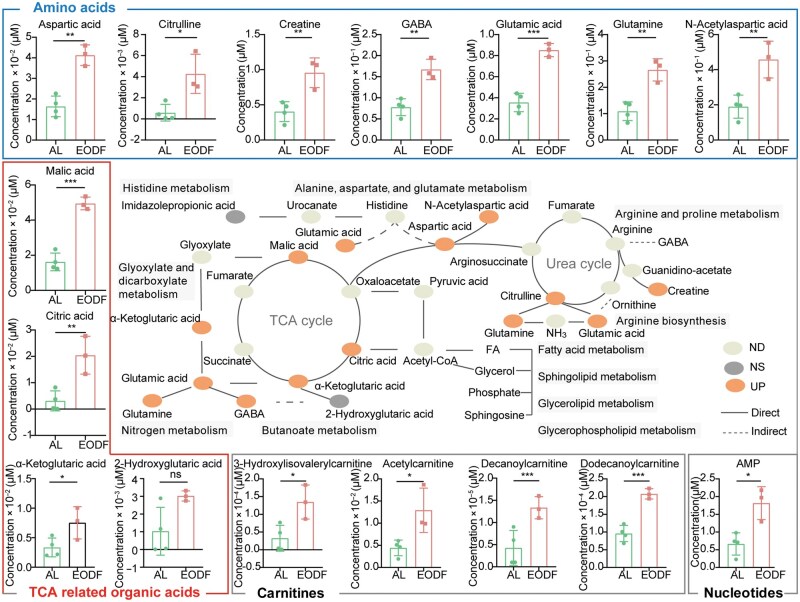
Schematic summary of metabolomics results for significantly enriched KEGG pathways in the EODF and AL groups. The peripheral histograms show raw concentration values (mean ± SD) of metabolites in EODF and AL samples from the different biological replicates. The central scheme shows the involvement of the metabolites in key metabolic pathways. *n *= 4 for AL group; *n *= 3 for EODF group. *P-*value: ^*^P < 0.05, ^**^  *P* < 0.01, ^***^  *P* < 0.001. ns, not significant.

Building on the TCA cycle-centric findings in Fig. 2, we constructed a global metabolic network integrating all the enriched pathways from KEGG analysis (Fig. 3). This system-level visualization reveals how these core TCA nodes and amino acids are embedded within a larger adaptive network, orchestrating carbon-nitrogen coordination across multiple pathways. Under starvation, mice maintain energy and metabolite homeostasis through multilevel metabolic reprogramming, during which a range of metabolites of lysosomal origin are significantly altered. Key metabolites of the TCA cycle show characteristic elevations. Among them, malic acid, together with citric acid and α-KG, integrates multiple metabolic pathways via the aspartate node. Elevated malic acid and α-KG achieve metabolic shunting by participating in glyoxylate and dicarboxylate metabolism. Malic acid promotes lipid metabolism through the oxaloacetate–pyruvate–acetyl-CoA pathway on the one hand, and maintains the balance of intermediates in the TCA cycle through the backfill reaction on the other hand. Meanwhile, α-KG together with citric acid regulates TCA cycle flux and lipid precursor supply through the acetyl-CoA node. These metabolites further bridge alanine, aspartate, and glutamate metabolism via the aspartate node, influence histidine metabolism, and enter the urea cycle via arginosuccinate, which regulates arginine and proline metabolism and arginine biosynthesis to maintain nitrogen homeostasis. This fine-tuned regulatory network dynamically balances gluconeogenesis, lipid metabolism, and amino acid homeostasis during energetic crises. This adaptive capacity is closely linked to the lysosomal metabolic context, suggesting that the autophagy-lysosome system provides key substrates and a regulatory basis for metabolic reprogramming during nutrient deprivation. The peripheral bar graphs in Fig. 3 compare the abundance of 16 metabolites between the EODF and AL groups. They all show significantly elevated levels in the EODF group, a phenomenon that may be associated with enhanced lysosomal metabolic activity and autophagic flow.

### Lysosome-associated lipids and TCA cycle reprogramming synergistically mediate metabolic adaptation under starvation

**Figure 4:**
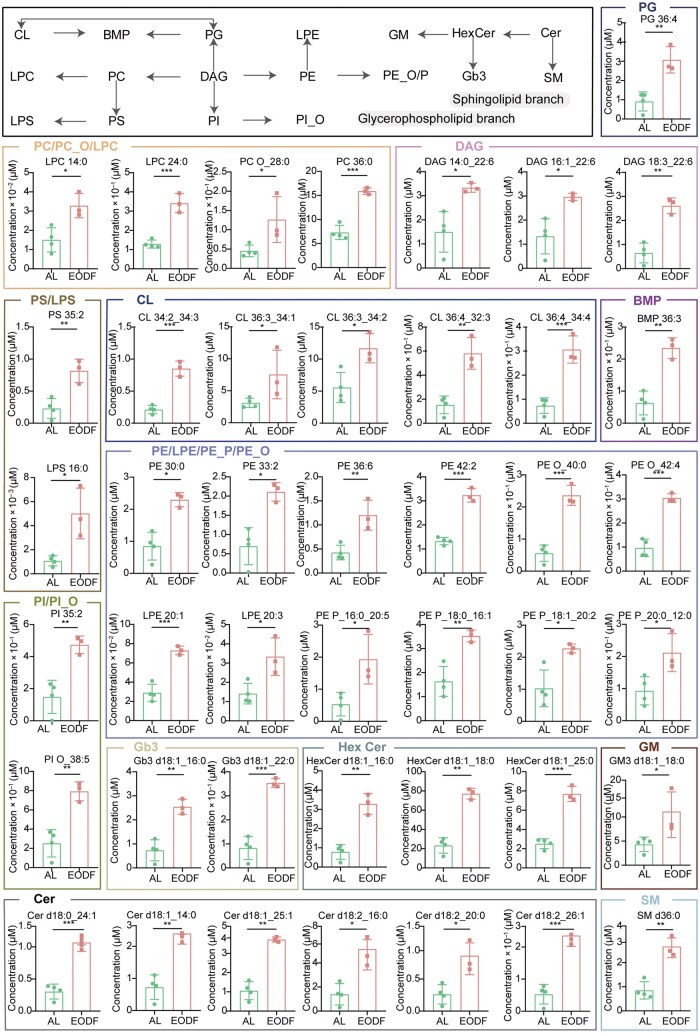
Schematic summary and quantitative analysis of significantly up-regulated lipids in the EODF and AL groups. *n *= 4 for AL group; *n *= 3 for EODF group. Data are presented as mean ± SD. *P-*value: ^*^P < 0.05, ^**^P < 0.01, ^***^P < 0.001.

The above analysis suggests that nutrient deprivation induced metabolic reprogramming of the TCA cycle (Fig. 3), which may in turn affect the levels of various types of lipids. Lipidomic analysis showed that 43 lipid molecules were significantly up-regulated in the EODF group when compared with the AL group (Fig. 4). These lipids can be classified into three major categories: glyceroli­pids, glycerophospholipids, and sphingolipids (Figs. 2d, 2e, and 4). Although some secondary lipids are not included in the KEGG database, their superior classifications are all covered by KEGG. Accordingly, based on the KEGG pathway database, we found that these upregulated lipids were primarily involved in glycerolipid metabolism, glycerophospholipid metabolism, and sphingolipid metabolism. Correlation clustering analysis demonstrated significant positive correlations between these lipids and various metabolites, including malic acid, citric acid, α-KG, glutamate, and aspartate (Supplementary Fig. S1). Moreover, malic acid, ­citric acid, and aspartate can all indirectly promote the synthesis of fatty acids, as well as glycerolipids and glycerophospholipids such as phosphatidylcholine (PC) and phosphatidylethanolamine (PE), through the acetyl-CoA node. Among these, the increase in sphingolipids, such as ceramide (Cer) and sphingomyelin (SM), suggests cellular membrane lipid remodeling. Notably, the significant increase in BMP, a lysosome-specific phospholipid, in the EODF group may be associated with the activation of the autophagy-lysosome system and enhanced membrane remodel­ing processes. These findings reveal a dynamic coupling ­mechanism between TCA ­cycle metabolic reprogramming and lipid metabolism under starvation conditions (Figs. 3 and 4), ­further expanding the metabolic network illustrated in Fig. 3. This indicates that energy homeostasis is maintained by coordinating carbon source allocation and lipid metabolic remodeling, a process potentially dependent on the recycling of lipids via the autophagy-lysosome system. These insights provide a new perspective for understanding the metabolic regulation of energy stress adaptation.

### Integrated multi-omics analysis identifies TCA-related metabolites and enzymes as central hubs for response under starvation conditions

**Figure 5:**
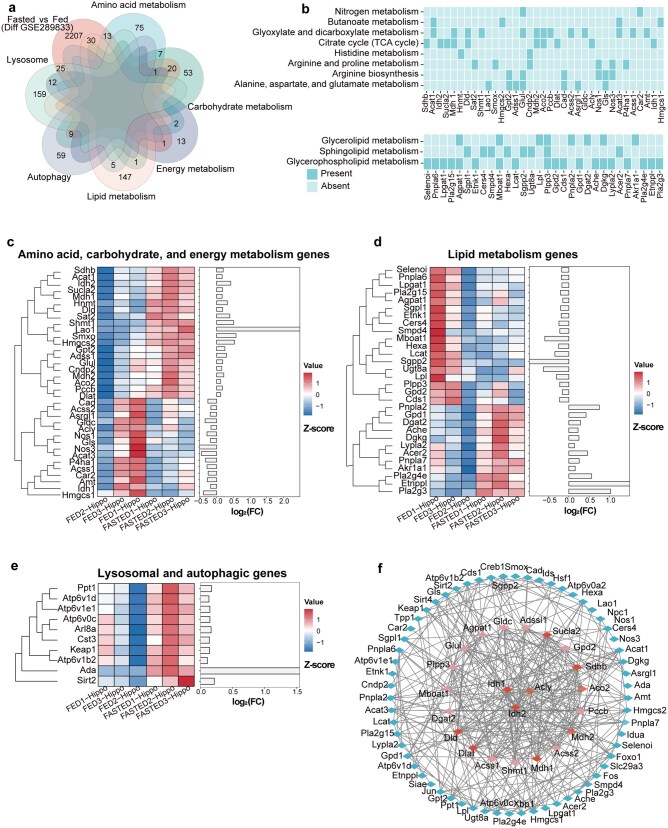
Transcriptomics analysis reveals dysregulated TCA cycle-related genes in fasted mice. (a) Multivariate Venn diagram analysis of differentially expressed genes from public transcriptomics data (Fasted versus Fed), including metabolic pathway genes (amino acid, carbohydrate, energy, and lipid metabolism), lysosomal genes, and autophagic genes. (b) Binary plots of pathways versus genes. The darker color means that the gene is in the pathway; the lighter color means that the gene is not included in the pathway. (c–e) Heatmaps showing the significantly different expression profiles of amino acid, carbohydrate, and energy metabolism genes (c), lipid metabolism genes (d), and lysosomal and autophagic genes (e) in fed and fasted mice. (f) Protein-protein interaction network analysis of the proteins encoded by all the significantly differentially expressed amino acid, carbohydrate, energy, lipid, lysosomal, and autophagic genes. *n *= 4 for AL group; *n *= 3 for EODF group. Highlighted nodes represent the core hub proteins and TCA cycle-related proteins identified by topological algorithms (Degree).

Next, we incorporated a public transcriptomic dataset (GSE289833) to investigate the transcriptional regulation mediating TCA cycle metabolic reprogramming. Differential expression analysis of this dataset, comparing hippocampal tissues from 18-h-fasted and normal-diet-fed mice, identified 2272 significantly altered genes (*P *< 0.05). To precisely establish functional linkages between transcriptomic changes and metabolomic/lipidomic profiles, we intersected these differentially expressed genes with gene sets (202 genes) from the eight significantly enriched metabolic pathways in Fig. 2f, 183 lipid metabolism-related genes (including glycerolipid, glycerophospholipid, and sphingolipid metabolism), 68 lysosomal genes, and 196 autophagy-associated genes. This integrated analysis yielded 99 overlapping genes (Fig. 5a), comprising 35 intersecting with the 8 significantly enriched metabolic pathways, 30 associated with lipid metabolism, and 34 linked to lysosomal and autophagy processes. A binary plot visually contextualizes these genes within their respective pathways (Fig. 5b). Further characterization of expression patterns uncovered a sophisticated transcriptional regulatory landscape. Visualization through combined heatmaps and log_2_(FC) bar plots clearly delineates gene-specific expression trends between groups (Fig. 5c–e). Notably, Fig. 5e specifically displays only those genes that were upregulated in the fasted group. Notably, TCA cycle-associated genes exhibited a finely coordinated regulatory pattern; for instance, *Idh2* was significantly up-regulated, whereas its isozyme *Idh1* was markedly down-regulated, suggesting a potential shift in metabolic flux direction. Protein–protein interaction (PPI) network analysis of the products of these 99 overlapping genes revealed that despite their differential expression, key TCA cycle enzymes including Idh1, Idh2, and acly function as central hubs within the network, with other TCA cycle enzymes also displaying high connectivity (Fig. 5f). This finding underscores the pivotal role of the TCA cycle at the protein interactome level. Together, these results demonstrate that despite originating from different data modalities (lysosomal metabolites versus tissue transcriptomes) and quantification approaches, the transcriptional adaptive reprogramming in hippocampal tissue under similar experimental conditions exhibits strong functional concordance with lysosomal metabolic changes. This multi-level integration elucidates a central mechanism wherein precise regulation of the TCA cycle enables metabolic adaptation to starvation stress.

### Diverse sources of lysosomal TCA-related metabolites during nutrient deprivation

**Figure 6:**
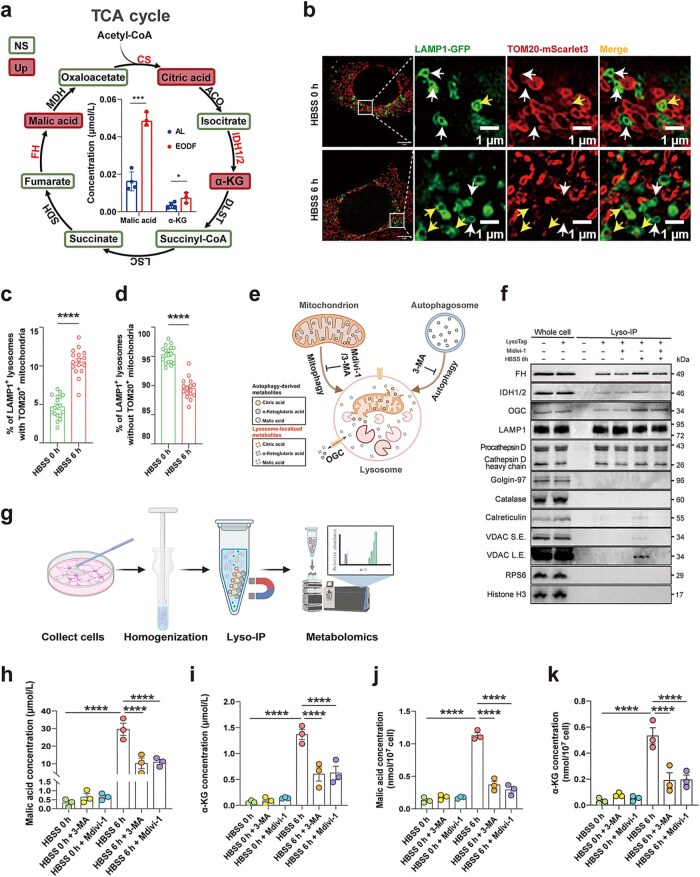
TCA cycle intermediates derived from non-autophagic sources are present within lysosomes. (a) Schematic overview of Lyso-IP-based metabolomic analysis of the TCA pathway in mouse hippocampal tissue. Differentially abundant metabolites in EODF lysosomes compared to AL lysosomes are highlighted in red brown. NS, not significant; UP, significantly upregulated. Bar plots show the concentrations of malic acid and α-KG in hippocampal lysosomes isolated from AL and EODF mice. Data are presented as mean ± SEM. (*n *= 4 for AL group; *n *= 3 for EODF group). (b–d) Representative SIM images and quantification of mitophagy in HT22 cells expressing LAMP1-GFP (lysosomes) and TOM20-mScarlet3 (mitochondria). Cells were starved in HBSS for 6 h, and LAMP1/TOM20 colocalization was quantified. At least 15 cells were analyzed per condition. Boxed areas in the left images are enlarged at the right. The white arrows indicate lysosomes (LAMP1^+^) that do not contain mitochondria (TOM20^−^), while the yellow arrows indicate lysosomes (LAMP1^+^) that contain mitochondria (TOM20^+^). Scale bar, 1 μm. (e) Schematic illustration depicting potential origins of TCA cycle intermediates within lysosomes. Created with BioRender.com. (f) Immunoblot analysis of lysosomes isolated from HT22 cells using antibodies against the indicated proteins. HT22 cells were subjected to HBSS-induced starvation for 6 h, with or without Mdivi-1 treatment. Blots are representative of three independent experiments. (g) Schematic diagram of extraction of lysosomes from HT22 cells and subsequent analysis of lysosomal metabolites. Created with BioRender.com. (h and i) Metabolomics analysis of malic acid and α-KG levels in lysosomes from HT22 cells. (j and k) Detection of malic acid and α-KG concentrations in HT22 lysosomes using kits. Data are presented as mean ± SEM. Statistical analysis was performed using the two-tailed unpaired Student’s *t*-test or one-way/two-way ANOVA, as appropriate. ^*^P < 0.05, ^**^P < 0.01, ^***^P < 0.001, ^****^P < 0.0001.

The roles of lysosomes in amino acid and lipid metabolism are well established. Under nutrient deprivation, lysosomal proteases, lipases, and phosphatases contribute to the degradation and recycling of proteins and lipids. However, their involvement in carbohydrate metabolism remains poorly defined [34–36]. Here, by performing Lyso-IP–based metabolomics, we found that EODF markedly increased the abundance of TCA cycle intermediates, including malic acid, citric acid, and α-KG, in mouse hippocampal lysosomes (Fig. 6a; Supplementary Fig. S2a). To explore the origin of these metabolites, we expressed LAMP1-GFP and TOM20-mScarlet3 in HT22 cells to visualize the lysosomes and the mitochondria, respectively, and then we starved the cells in Hank’s balanced salt solution (HBSS) for 6 h. Nutrient deprivation progressively increased the colocalization of lysosomes with mitochondria (Fig. 6b and c), and we similarly observed expression of the mitochondrial marker VDAC in the lysosomes of HT22 cells under the HBSS-6h condition in our western blotting results (Fig. 6f), consistent with previous studies [37, 38]. These results suggest that, under starvation, mitochondrial components are more readily delivered to the lysosomes for degradation, and that mitochondria-derived metabolite input may, at least in part, contribute to the accumulation of TCA cycle intermediates within lysosomes. Notably, a substantial proportion of lysosomes did not exhibit detectable mitochondrial components (Fig. 6b and d), and malic acid displayed relatively high basal levels in lysosomes even under AL conditions (Fig. 6a). This observation suggests that the establishment of a lysosomal pool of TCA-related metabolites is not necessarily entirely dependent on mitochondrial input. We propose two non-mutually exclusive mechanisms: (i) local gene­ration and/or interconversion of TCA-related metabolites within the lysosomal lumen and (ii) synthesis of these metabolites predominantly in the mitochondria, followed by EODF-induced redistribution into lysosomes via specific transport pathways, leading to their lysosomal enrichment (Fig. 6e).

Non-mitochondrial localization of TCA-related enzymes has been widely reported [39–41]. Given the significant increase of multiple TCA cycle intermediates in lysosomes under starvation, we hypothesized that their accumulation might not solely depend on mitochondria–lysosome crosstalk/communications but could also involve potential TCA-related enzymatic activities within lysosomes. Following 6 h of starvation, VDAC became detectable in lysosomes and the abundance of IDH1/2 and FH increased, suggesting the involvement of mitochondria-associated lysosome-dependent pathways. Nevertheless, FH and IDH1/2 signals persisted under basal conditions and upon treatment with the mitochondrial division inhibitor 1 (Mdivi-1), suggesting that a subset of these enzymes may exist as resident lysosomal components (Fig. 6f; Supplementary Fig. S2h and i). In contrast, citrate synthase (CS) was detected only after 6 h of starvation and was abolished by Mdivi-1, indicating that its lysosomal presence is strongly associated with starvation-induced delivery of mitochondria-related components into lysosomes (Supplementary Fig. S2d and j).

To assess the generality of the increase in lysosomal TCA-related metabolites and to exclude potential tissue-specific effects, we established an *in vitro* Lyso-IP workflow in HT22 cells (Fig. 6g). Under HBSS-induced starvation, metabolomic profiling of Lyso-IP isolates revealed a significant elevation of lysosomal malic acid, α-KG, and citric acid (Fig. 6h and i; Supplementary Fig. S2b). Notably, 3-methyladenine (3-MA) or Mdivi-1 did not completely reverse the starvation-induced accumulation of these metabolites in lysosomes. These results suggest that enrichment was not solely dependent on canonical autophagy or mitophagy. We acknowledge the limitations of using 3-MA, which primarily targets class III PI3K (VPS34) to suppress autophagy initiation, but 3-MA can also affect endosomal maturation, lysosomal trafficking, endo-lysosomal functions, and class I PI3Ks [42, 43]. Thus, the phenotypes observed after 3-MA treatment cannot be attributed exclusively to autophagy inhibition. Despite these caveats, the starvation-induced increase in lysosomal TCA-related metabolites was consistently observed. We further validated these findings by quantifying malic acid, α-KG, and citric acid in Lyso-IP samples using assay kits, which yielded results consistent with the metabolomic profiling (Fig. 6j and k; Supplementary Fig. S2c).

**Figure 7:**
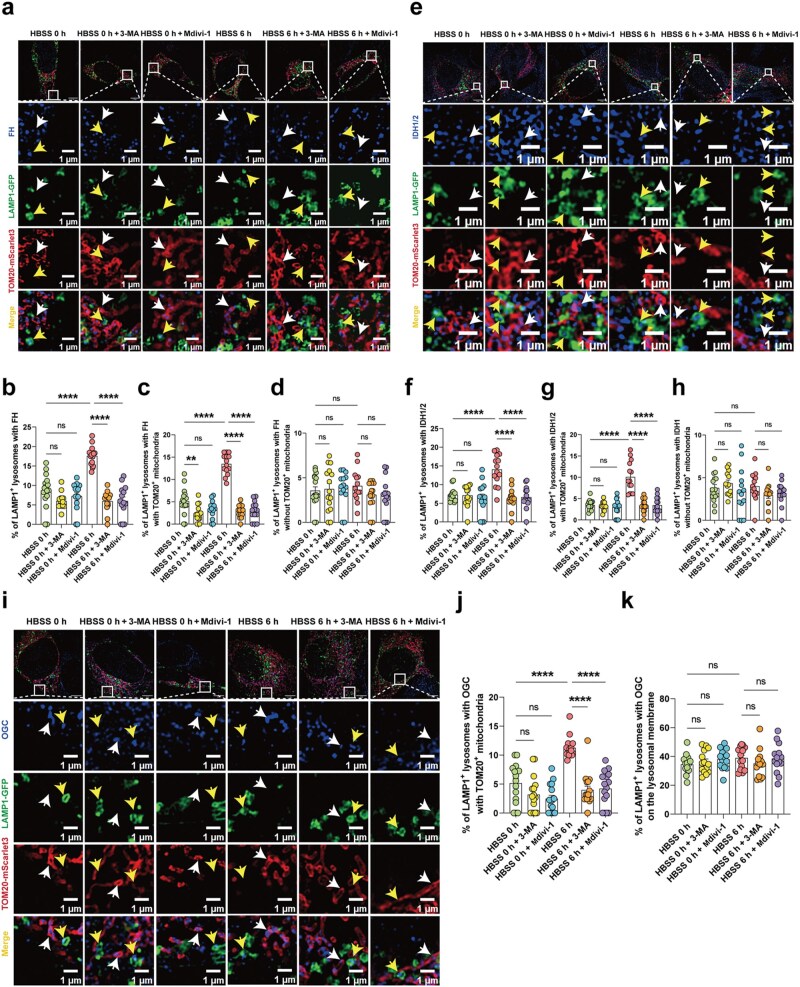
FH, IDH1/2, and the transporter OGC are localized to lysosomes. (a–d) Representative images of the subcellular localization of FH in HT22 cells (a). Yellow arrows indicate colocalization of the lysosomes with FH, whereas white arrows indicate colocalization of the mitochondria with FH. Quantification of FH signals in total LAMP1^+^ lysosomes, in LAMP1^+^ TOM20^+^ compartments representing mitophagy-associated pools, and in LAMP1^+^ TOM20^−^ lysosomes representing non-mitophagic pools (b–d). At least 15 cells were analyzed per condition. Scale bar, 1 μm. (e–h) Representative images of the subcellular localization of IDH1/2 in HT22 cells (e). Yellow arrows indicate colocalization of lysosomes with IDH1/2, and white arrows indicate colocalization of the mitochondria with IDH2. Quantification of IDH1/2 signals in total LAMP1^+^ lysosomes, in LAMP1^+^ TOM20^+^ compartments representing mitophagy-associated pools, and in LAMP1^+^ TOM20^−^ lysosomes representing non-mitophagic pools (f–h). At least 15 cells were analyzed per condition. Scale bar, 1 μm. (i–k) Representative images of the subcellular localization of OGC in HT22 cells (i). Yellow arrows indicate OGC on the lysosomal membrane, whereas white arrows indicate OGC on the mitochondrial membrane. Quantification of OGC on the LAMP1^+^ lysosomal membrane and on the TOM20^+^ mitochondrial membrane (j and k). At least 15 cells were analyzed per condition. Scale bar, 1 μm. Endogenous proteins (blue) were detected by immunofluorescence. Lysosomes were labeled with LAMP1-GFP (green) and the mitochondria were labeled with TOM20-mScarlet3 (red). Data are presented as mean ± SEM. Statistical analysis was performed using two-tailed unpaired Student’s *t*-test or one-way/two-way ANOVA, as appropriate. ^*^P < 0.05, ^**^P < 0.01, ^***^P < 0.001, ^****^P < 0.0001.

To further validate these findings, we examined the subcellular localization of CS, FH, and IDH1/2 in HT22 cells by immunofluorescence. HT22 cells were cultured under normal conditions or subjected to HBSS-induced starvation for 6 h, with or without additional treatment with Mdivi-1 or 3-MA. Cells were co-transfected with LAMP1-GFP and TOM20-mScarlet3 to label lysosomes and mitochondria, respectively. Interestingly, they also exhibited colocalization with the lysosomal marker LAMP1 (Fig. 7a, b, e, and f; Supplementary Fig. S2e and f). Quantitative analysis revealed that starvation increased the proportion of CS, FH, and IDH1/2 signals in LAMP1^+^ TOM20^+^ compartments, and this effect was reversed by Mdivi-1 or 3-MA (Fig. 7c and g; Fig. S2f), indicating the involvement of mitochondria-associated lysosome-dependent trafficking mechanisms. Notably, FH and IDH1/2 were also detected in LAMP1^+^ TOM20^−^ lysosomes, irrespective of starvation and Mdivi-1 or 3-MA treatment (Fig. 7d and h), suggesting their constitutive presence as resident lysosomal enzymes. In contrast, CS was not observed in LAMP1^+^ TOM20^−^ lysosomes (Supplementary Fig. S2g). These observations suggest a potential role for FH and IDH1/2 in lysosomal TCA metabolism, although functional contribution remains to be determined.

In parallel, we assessed the oxoglutarate carrier (OGC, SLC25A11) in Lyso-IP–isolated lysosomal fractions by western blotting. Under basal conditions, OGC was readily detectable in lysosomes. Following HBSS-induced starvation, lysosome-associated OGC levels increased, whereas 3-MA/Mdivi-1 partially attenuated this elevation (Fig. 6f; Supplementary Fig. S2k). To further substan­tiate these findings, we next performed immunofluorescence ana­lysis to evaluate the subcellular localization of OGC. As expected, OGC was strongly localized to the mitochondria. Notably, it also exhibited distinct colocalization with LAMP1^+^ lysosomes, irrespective of starvation and Mdivi-1 or 3-MA treatment (Fig. 7i–k), suggesting its potential localization to lysosomes. Together with the detection of FH and IDH1/2, these observations suggest that malic acid and α-KG in lysosomes may originate from both resident enzymatic activity and putative OGC-mediated transport. Under nutrient deprivation, such complementary inputs may contribute to lysosomal metabolite turnover, although their precise functional relevance remains to be determined.

## Discussion

In this study, we establish an *in vivo* AAV-LysoTag/Lyso-IP workflow in mouse hippocampus (with parallel cell-based validation) to resolve lysosomal metabolites and lipids [40]. First, we map a reprogramming of the hippocampal lysosomal metabolite–lipid landscape under EODF conditions, consistent with the view that lysosomes act as metabolic hubs coordinating degradation and nutrient signaling. Second, we uncover robust enrichment of TCA-related intermediates in hippocampal lysosomes and identify a subset of TCA enzymes therein. Together with evidence of altered mitochondria–lysosome crosstalk and lysosomal localization of transporters, these data indicate that mitochondrial metabolite input, resident enzymatic activity, and carrier-mediated exchange jointly sustain a lysosomal TCA cycle-related pool during nutrient stress. Third, by deploying Lyso-IP–based profiling alongside imaging and immunoblotting, we provide an integrated hippocampal resource that links lysosomal composition to neuronal metabolic demands, thereby reframing lysosomes from terminal degradative endpoints to adaptable metabolic nodes in the central nervous system (CNS).

The capacity of lysosomes to withstand stress plays an important role in neuronal metabolism under nutrient deprivation. Under such conditions, lysosomal catabolism becomes a ­significant source of metabolic substrates [3]. Interestingly, intermittent fasting has been shown to improve brain functions such as cognition and synaptic plasticity [44]. During the energy stress induced by EODF, the autophagy-lysosome degradation pathway is enhanced, delivering a large number of cellular components to lysosomes for hydrolytic degradation [45, 46]. The resulting amino acids, fatty acids, and other small metabolites can be reutilized to meet cellular energy and biosynthetic demands. For example, Jaishy *et al*. reviewed how lysosomes recycle intracellular lipids via autophagy, supplying breakdown products for cellular energy and biosynthesis [45]. Abu-Remaileh *et al*. reported that under nutrient deprivation or mTORC1 inhibition, lysosomal efflux of essential amino acids is significantly reduced, effectively turning lysosomes into amino acid reservoirs [5]. Therefore, EODF may lead to the accumulation of amino acids such as glutamate, glutamine, and aspartate in lysosomes, providing a reserve of available amino acids during energy shortages. At the same time, autophagy also promotes lipolysis (lipophagy), releasing free fatty acids for β-oxidation [45]. The observed increases in medium-chain acylcarnitines (octanoylcarnitine, lauroylcarnitine, and 3-hydroxyisovalerylcarnitine) may reflect enhanced lysosome-mediated fatty acid mobilization. The enrichment of polyunsaturated fatty acids (e.g. adrenic acid) may be related to the breakdown of membrane phospholipids. The accumulation of these metabolites in lysosomes suggests that neurons “pre-store” energy substrates during fasting: elevated AMP can activate AMP-activated protein kinase (AMPK), which further inhibits mTORC1 and promotes autophagy [45], forming a feedback loop that maintains energy homeostasis. In summary, the integration of these metabolic cycles and lysosome-mitochondrion cooperation collectively shapes the profile of metabolites enriched in lysosomes under fasting conditions.

In our lipidomic analysis, EODF hippocampal lysosomes exhibited upregulation of various phospholipids and sphingolipids, closely associated with enhanced autophagy-lysosome activity and membrane lipid turnover [46–48]. During autophagy, damaged mitochondria, organelle membranes, and synaptic membranes are engulfed and delivered to lysosomes, releasing abundant lipid components for degradation. BMP is a signature phospholipid of lysosomal internal vesicles (ILVs), providing a platform for lipid hydrolases and transporters to facilitate degradation and sorting of sphingolipids, glycerophospholipids, and neutral lipids [49, 50]. The observed elevation of BMP may reflect increased ILV formation to process excess lipids delivered by autophagy or endocytosis. Consistent with this, recent studies have found that BMP promotes lipid degradation and sorting in lysosomes and is frequently dysregulated in lysosomal storage disorders and neurodegenerative diseases [49–51]. Thus, upregulation of BMP after EODF may indicate enhanced lysosomal lipid turnover—an adaptive mobilization of stored lipids [35, 36]. Regarding sphingolipids, we found signi­ficant enrichment of various ceramides, hexosylceramides, and sphingomyelins. These complex glycosphingolipids are abundant in the nervous system and serve as major components of myelin and neuronal membranes. BMP is closely linked to sphingolipid metabolism and promotes the binding of sphingolipid activator proteins to intraluminal membranes, presenting cholesterol and sphingolipids to degradative enzymes [47]. Thus, the increase in sphingolipids suggests intensified breakdown of membrane glycosphingolipids and an active lysosomal response to process them. Moreover, ceramides themselves act as cellular stress signaling molecules that can promote autophagy [52]. In terms of phospholipids, the increase in CL is particularly noteworthy. CL, the main phospholipid of the inner mitochondrial membrane, is involved in regulating mitochondrial dynamics and function. The elevation of CL suggests enhanced mitophagy. Chu *et al*. reported that va­rious autophagy stimuli (such as rapamycin) in neuronal cells can induce CL externalization and promote LC3-mediated mitophagy [53]. Therefore, the increase in CL in hippocampal lysosomes may reflect EODF-induced mitophagy to meet energy metabolism and stress adaptation needs. We also observed elevated levels of phosphatidylinositol (PI), PE, and their ether forms, which may be associated with autophagosome membrane formation and defense against oxidative stress. PE plasmalogens have antioxidant properties and protect membranes under oxidative stress [54]. A recent study found that in the early stages of an Alzheimer’s disease model, mice transiently upregulate ethanolamine plasmalogens to help clear reactive oxygen species and promote phagocytosis [54]. In our study, certain ethanolamine plasmalogen species (e.g. PE O-42:4, PE O-40:0, and PE P-18:0_18:0) were increased, possibly representing a protective response to fasting-induced metabolic stress. Additionally, increases in common membrane phospholipids such as PC and phosphatidylserine (PS) reflect the recycling and synthetic balance of membrane components. Notably, lysosomal hydrolysis of phospholipids produces intermediates such as diacylglycerol (DAG) and lysophospholipids, including lysophosphatidylcholine, lysophosphatidylethanolamine, and lysophosphatidylserine. These small molecules can act as sig­naling lipids, be re-esterified for phospholipid synthesis, or serve as energy substrates; thus, their increased levels are consistent with active lysosomal membrane lipid metabolism. Overall, these lipidomic changes suggest that hippocampal neurons adapt to EODF-induced nutrient stress by modulating lysosomal lipid metabolism. This highlights the significance of further studying the roles of autophagy and lipid homeostasis in neuroprotection and metabolic diseases [35, 36, 49, 50].

Interestingly, our *in vivo* metabolomic analysis revealed that EODF significantly increased the levels of malic acid, citric acid, and α-KG in hippocampal lysosomes. Multiple pathways may contribute to this enrichment of TCA intermediates. One major source of these intermediates is selective crosstalk: under metabolic stress, damaged or excess mitochondria are engulfed by autophagosomes and fused with lysosomes, releasing their contents into the lysosomal lumen and naturally enriching lysosomal TCA intermediates and enzymes. Our *in vitro* experiments (HBSS starvation for 3–6 h) confirmed an increased colocalization between LAMP1^+^ lysosomes and TOM20^+^ mitochondria, consistent with previous studies showing that nutrient stress induces mitophagy [55]. It should be emphasized that whether nutrient deprivation induces mitophagy is highly context dependent. In multiple acute starvation paradigms, the mitochondria undergo elongation/­hyperfusion, which reduces their likelihood of being captured by autophagosomes and helps sustain energy output [37, 56]. However, starvation can also trigger mitophagy under specific metabolic cues. Zhang *et al*. reported that short-term fasting initiates mitophagy via a cascade in which a decrease in cytosolic acetyl-CoA releases NOD-like receptor (NLR) family member X1 (NLRX1) autoinhibition and promotes LC3 binding, and that acetate supplementation reverses this process [57]. Importantly, although the starvation medium used in the study by Zhang *et al*. differed from the acute HBSS deprivation employed in our present study, we consistently observed the presence of mitochondrial components within lysosomes under HBSS conditions. Regardless of the multiple delivery routes, the HBSS-induced accumulation of these metabolites in lysosomes was significantly attenuated by 3-MA or Mdivi-1, indicating that mitochondrial input contributes to this process. In SH-SY5Y cells, HBSS treatment also leads to colocalization of LC3 puncta with mitochondrial signals, suggesting that starvation recruits the autophagy machinery to the mitochondria and engages the autophagy pathway [58]. Notably, the delivery of mitochondrial contents to lysosomes is not restricted to canonical mitophagy. Non-canonical pathways, including mitochondria-derived vesicles [59, 60], mitochondria-lysosome contact sites [61], and microautophagy-like processes [62], can also mediate the transfer of mitochondrial components to lysosomes with varying degrees of scale and selectivity. However, two observations suggest that mitochondrial delivery to lysosomes alone cannot explain all the findings: (i) malic acid has a high baseline level in lysosomes even under AL conditions and (ii) a large number of LAMP1^+^/TOM20^−^ lysosomes are also enriched in TCA intermediates. Previous studies have shown that certain cytosolic metabolic enzymes can be recognized by chaperones such as heat shock cognate protein 70 (HSC70) and delivered to lysosomes via lysosome-associated membrane protein type 2A (LAMP2A)-mediated chaperone-mediated autophagy [63]. Moreover, a *Drosophila* study reported that during starvation, lysosomes convert cystine into acetyl-CoA and feed it into the TCA cycle to restrict cell growth [30], indicating that lysosomal metabolic outputs can modulate the cellular response to nutrient deprivation. Combining HT22-Lyso-IP western blotting and immunofluorescence, we detected lysosomal signals of IDH1/2 and FH under both basal and starvation conditions. In contrast, CS appeared in lysosomes only after 6 h of starvation and was abolished by Mdivi-1 treatment, indicating that lysosomal localization of CS depends on mitochondria–lysosome crosstalk. Conversely, IDH1/2 and FH appear to be at least partially resident in lysosomes. There are three IDH isoforms: cytosolic/peroxisomal NADP^+^-dependent IDH1, mitochondrial NADP^+^-dependent IDH2, and a mitochondrial NAD^+^-dependent heterotetramer, IDH3 [64, 65]. IDH1 and IDH2 share approximately 70% sequence identity in humans [66] and catalyze the same isocitrate-to-α-KG reaction; their main difference is localization (IDH1 in the cytosol/peroxisomes, and IDH2 in the mitochondria) [65]. This sequence similarity explains why common IDH1 antibodies often cross-react with IDH2. Considering reports of non-mitochondrial localization of various TCA enzymes [39–41], we speculate that partial TCA reaction sequences may occur in or near lysosomes. For example, IDH1/2 catalyzes the oxidative decarboxylation of isocitrate to α-KG, while FH catalyzes the reversible hydration of fumarate to malic acid [39–41, 55]. It has been found that amino acids mobilized by autophagy and glutamine metabolism in the cytosol generate α-KG, which can be transported into the mitochondria via SLC25A11 to replenish the TCA cycle and support ATP production [67, 68]. Interestingly, we observed a signal in the LAMP1^+^ compartments consistent with OGC [67], suggesting that there may also be an α-KG/malic acid exchange across the lysosomal membrane. From these observations, we propose a model in which the lysosomal metabolite profile is reshaped by three parallel pathways: (i) input mediated by mitochondrial delivery to lysosomes; (ii) local generation by resident enzymes; and (iii) transporter-mediated substrate exchange. Under nutrient deprivation, the lysosome-autophagy pathway becomes a crucial source of substrates and a metabolic hub for neurons [35, 36]. On one hand, lysosome-derived α-KG, malic acid, and citric acid can serve as “fill-in” substrates to supplement the mitochondrial TCA cycle, maintaining ATP production and anabolic metabolism. On the other hand, metabolites such as α-KG, 2-hydroxyglutarate, and succinate have signaling properties and may regulate mTORC1/AMPK and dioxygenase activities at the lysosome-cytosol interface, thus participating in global metabolic reprogramming under energy stress [5, 34]. In a high-demand region like the hippocampus with limited metabolic reserve, a “lyso­somal pool of TCA intermediates” can be considered an adaptive reserve: when external energy supply is limited, lysosomes export and redistribute metabolites to provide neurons with urgently needed carbon skeletons and regulatory signals, thereby delaying energy failure and enhancing metabolic resilience.

Overall, in the context of “brain nutrient deprivation”, our hippocampal Lyso-IP analysis depicts a lysosomal TCA metabolite pool driven by mitochondrial delivery to lysosomes, local production by resident enzymes, and transporter-mediated exchange. Our results reveal that EODF-associated lysosomal lipid reprogramming (upregulation of BMP, ceramides, and DAG) may support energy and membrane homeostasis. Together with recent high-quality stu­dies, these results extend the concept of lysosomes as metabolic integration hubs to the level of the CNS, providing new mechanistic insights and methodological approaches for understanding hippocampal metabolic resilience under energy stress.

### Limitations of the study

There are several limitations in our present study. First, although we identified the presence of TCA cycle-related enzymes within the lysosomal compartment, we did not perform *in situ* enzyme activity assays. Consequently, the catalytic competence of enzymes such as IDH1/2 or FH within the acidic microenvironment of the lysosome remains to be definitively established. Second, our determination of protein localization relied primarily on fractionation-western blotting and structured illumination fluorescence microscopy (SIM). These methods may not fully resolve the precise sub-organellar distribution; thus, further validation via immuno-electron microscopy is required to conclusively distinguish between enzyme localization on the lysosomal membrane, within the intraluminal vesicles, or at mitochondria-lysosome contact sites. Third, our mechanistic inferences regarding metabolite enrichment are currently based on observational associations rather than causal intervention. Genetic ablation or specific manipulation of candidate transporters (e.g. OGC/SLC25A11) is necessary to firmly establish their causal roles in shaping the lysosomal TCA cycle metabolite profile. Fourth, although we detected TCA cycle metabolites in lysosomes, our data reflects steady-state abundance. Future studies employing stable isotope tracing or metabolic flux analysis are essential to quantify the relative contributions of *in situ* lysosomal synthesis versus metabolite transportation. Finally, our reliance on pharmacological inhibitors (3-MA and Mdivi-1) to perturb mitochondria-lysosome crosstalk carries inherent limitations regarding specificity. As these compounds can exert off-target effects on broad endo-lysosomal or mitochondrial processes, the observed phenotypes should be interpreted within this methodological context, and future validation using genetic models is warranted to confirm these findings.

## Materials and methods

### Reagents and antibodies

Detailed information on the antibodies used in this study is provided in Supplementary Table S1. Additional reagents included goat anti-rabbit Alexa Fluor 647 secondary antibody (1:1000, #4414) for immunofluorescence from Cell Signaling Technology; HRP-conjugated goat anti-rabbit IgG (H + L) secondary antibody (1:5000, #BF03008) for western blotting from Biodragon; Dulbecco’s modified Eagle medium (DMEM, C11965500BT), HBSS (14175079), Opti-MEM (31985070), Lipofectamine 2000 (11668019), fetal bovine serum (FBS, A5256701), and an ECL chemiluminescent substrate kit (32109) from Thermo Fisher Scientific; penicillin–streptomycin (SV30010) from Hyclone; HA-Tag immunoprecipitation magne­tic bead kit (TB100028) from SinoBiological; Complete Protease Inhibitor Cocktail (11697498001) and PhosSTOP Phosphatase Inhibitor (4906845001) from Roche; mitophagy inhibitor Mdivi-1 (T1907) from TargetMol; autophagy inhibitor 3-MA (HY-19312) from MedChemExpress; and Antifade Mounting Medium with 4’,6-diamidino-2-phenylindole (DAPI) (P0131) from Beyotime.

### Plasmid construction and transfection

The plasmids pLJC5-TMEM192-3×HA and pLJC5-LAMP1-EGFP were constructed by dual restriction enzyme digestion and homologous recombination. Primers used for cloning were as follows: *TMEM192*-F: 5′-CCACCGGTACCATGGCGGCGGGGGGCAGGATGGAGGAC-3′; *LAMP1*-F: 5′-TTTTTTGTTAGACGAAGCGCTAGCATGGCGGCCCCCGGCG-3′; *TMEM192*-HA-R: 5′-CCGGAATTCTTAGCCGCTCCCTCCAGCATAATC-3′; *EGFP*-R: 5′-GTCTCGAGGTCGAGAATTCTTACTTGTACAGCTCGTCCAT-3′. The pTom20-mScarlet3-H plasmid was a gift from Dr. Zhifei Fu (Fujian Medical University). All plasmids were transiently transfected into HT22 cells using Lipofectamine 2000 according to the manufacturer’s instructions. Subsequent experiments were performed 24–48 h after transfection.

### AAV production and stereotaxic injection

An AAV expressing mouse Tmem192–EGFP–3×HA was produced for neuronal overexpression. Plasmids pHBAAV-mouse-Tmem192–EGFP–3×HA, pAAV-RC, and pHelper were co-transfected into HEK-293T cells using a lipid-based transfection method. Seventy-two hours post-transfection, cells were harvested, and AAV particles were purified (including removal of residual nucleic acids, column purification, and ultrafiltration concentration). The AAV titer was then determined using a standard assay.

For lysosome labeling *in vivo*, 8-week-old C57BL/6J mice were anesthetized with isoflurane and fixed in the stereotaxic apparatus (RWD). Bilateral hippocampal injections were performed using a glass micropipette. Stereotaxic coordinates were set according to a mouse brain atlas (relative to Bregma: anteroposterior, −2.0 mm; mediolateral, ± 1.5 mm; dorsoventral, −1.4 mm). A total volume of 0.5 μL of the AAV stock was injected per side at a rate of 0.1 μL/min. The needle was left in place for 5 min after injection before being slowly withdrawn, and the incision was sutured. After a 1-week postoperative recovery under standard husbandry, mice were assigned to either an EODF regimen or AL feeding for 8 weeks; EODF was defined as alternating 24-h fasting and 24-h feeding cycles.

### Experimental animals

This study utilized C57BL/6J mice bred in the animal facility at Shanghai Medical College of Fudan University. All mice were maintained in a controlled environment at 22 °C and approximately 50% humidity under a standard 12-h light/12-h dark cycle, with free access to food and water. To achieve lysosome-specific labeling in the hippocampus, AAV injection was carried out as described above. After a 1-week recovery period, the mice were randomly divided into two groups, AL group and EODF group, for a duration of 8 weeks. During this period, body weight was recorded at fixed time points (at each feeding/fasting switch) and general health was monitored. Water was provided *ad libitum* throughout. All procedures and husbandry were conducted in accordance with protocols approved by the Institutional Animal Care and Use Committee of Fudan University.

### Cell culture

Mouse hippocampal neuronal cells (HT22, TCM-C821, Haixing Biosciences) were maintained in DMEM supplemented with 10% FBS and 1% penicillin–streptomycin. Cells were cultured at 37 °C in a humidified incubator with 5% CO_2_. Mdivi-1 has been reported to inhibit dynamin-related protein 1 (Drp1)-mediated mitochondrial fission and is commonly used as a pharmacological tool to perturb mitochondrial dynamics and, to some extent, reduce Drp1-associated mitophagy [69]. 3-MA is a PI3K inhibitor that is classically used to suppress autophagy initiation/autophagosome formation [70]. For pharmacological inhibition, cells were pretreated for 3 h with either Mdivi-1 (10 μmol/L; to inhibit mitophagy) or 3-MA (1 mmol/L; to inhibit autophagy), and the corresponding inhibitor was maintained throughout the subsequent 6-h HBSS incubation.

### Immunofluorescence

For tissue immunofluorescence, mice were deeply anesthetized and transcardially perfused with 4% paraformaldehyde (PFA) for *in vivo* fixation. Brains were removed and post-fixed in 4% PFA at 4 °C for 4–6 h. Then cryoprotection was achieved by immersion in a sucrose gradient solution. Coronal brain sections (30-μm thickness) were prepared using a Frozen Section Machine (CM1950, Leica) for immunostaining. For cultured cells, cells on coverslips were fixed with 4% PFA at room temperature for 30 min. After fixation, tissue sections and cell samples were washed with PBS and permeabilized with 0.25% Triton X-100 for 30 min. Samples were then blocked with 10% FBS in PBS at room temperature for 2 h. Primary antibodies (Supplementary Table S1) were applied to samples, and incubation was carried out overnight at 4 °C in the dark. The next day, samples were washed with PBS and then incubated with Alexa Fluor 647-conjugated secondary antibodies for 1 h at room temperature in the dark. Coverslips were mounted with antifade mounting medium with DAPI. The fluorescent signals from TMEM192–EGFP–3×HA, pTom20-mScarlet3-H, and Lamp1-EGFP were detected directly as native fluorescence without antibody amplification. Fluorescence images were acquired using a Leica SP8 laser scanning confocal microscope or an Olympus spinSR structured illumination microscopy system (SIM-Ultimate, Olympus), using appropriate excitation/emission settings. To ensure comparability, identical laser power, detector gain, and exposure settings were used for all samples within the same experimental batch. All experiments were independently repeated at least three times, with multiple fields of view captured for each condition.

### Rapid isolation of lysosomes and metabolite extraction

For isolation of intact lysosomes, Lyso-IP was performed in HT22 cells and mouse hippocampal tissue following similar steps described in published Lyso-IP protocols [5, 6]. The background was subtracted using matched negative controls (HT22 cells transfected with plasmid vector, or mice injected with AAV-control). To maximize preservation of endogenous metabolites, all solutions were prepared using pre-chilled Optima-grade LC/MS water and all steps were performed on ice or at 4 °C. The buffer used for lysosome isolation was KPBS (136 mmol/L KCl and 10 mmol/L KH_2_PO_4_, adjusted to pH 7.25 with KOH). HT22 cells (approximately 3.5 × 10^7^) or mouse hippocampal tissue of equivalent weight was quickly rinsed with ice-cold PBS, resuspended in 1 mL KPBS, and then pelleted by centrifugation at 1000 *g* for 2 min at 4 °C. The pellet was gently resuspended in 950 μL KPBS, and a 25-μL aliquot (2.5% of the total volume) was set aside as the whole-cell input sample. The remaining suspension was homogenized on ice using a 2-mL Dounce homogenizer (20 gentle strokes) and centrifuged at 1000 *g* for 2 min at 4 °C. The post-nuclear supernatant, containing lysosomes and other organelles, was incubated with 150 μL of pre-washed anti-HA magnetic beads with gentle rotation for 15 min at 4 °C to capture HA-tagged lysosomes. The beads were then washed three times with cold KPBS (gentle mixing on a magnetic rack) to obtain purified lysosome fractions.

After the final wash, ice-cold metabolite extraction solvent was immediately added to both the lysosome bead pellet and the reserved whole-cell input sample. For the 25 μL input sample, 225 μL of extraction solvent (10-fold volume) was added. Samples were gently mixed to lyse the lysosomes and extract metabolites. The lysate mixtures were centrifuged at ≥ 20,000 *g* for 10 min at 4 °C. The resulting supernatants, containing extracted metabolites, were collected, snap-frozen in liquid nitrogen, and stored at −80°C until analysis.

### Western blotting

Western blotting was performed on both total lysates and lysosome immunoprecipitates (Lyso-IP samples) from lysosome-tagged (TMEM192–3×HA-expressing) and control mouse hippocampal tissues. Tissues were homogenized and lysed in RIPA buffer, and proteins were separated by SDS–PAGE (Epizyme Biotech) and transferred onto PVDF membranes. Membranes were blocked with 5% non-fat milk in TBST for 1 h at room temperature, and then incubated overnight at 4 °C with primary antibodies (Supplementary Table S1) diluted in TBST containing 5% BSA. The following day, membranes were washed three times in TBST (10 min each) and incubated with appropriate HRP-conjugated secondary antibodies for 1 h at room temperature. After washing, protein bands were visualized using an ECL chemiluminescent substrate and imaged with a gel documentation system (MiniChem610, SINSAGE).

### *In vitro* detection of malic acid, citric acid, and α-KG concentrations

HT22 cells were cultured in DMEM supplemented with 10% FBS and 1% penicillin-streptomycin at 37 °C under 5% CO_2_ until 80% confluence. Lysosomes were purified from the cells using the Lyso-IP method. The concentrations of malic acid, citric acid, and α-KG in the lysosomal fraction were measured using commercially available kits from Solarbio (BC2155 for malic acid, BC5490 for citric acid, and BC5425 for α-KG) according to the manufacturer’s instructions. The absorbance was measured using a microplate reader, and the metabolite concentrations were determined by comparing the readings to standard curves. All experiments were performed in triplicate, and the data are presented as the mean ± SD.

### Targeted metabolomics analysis

Targeted metabolite analysis was performed using an ultra-performance liquid chromatography–tandem mass spectrometry (UPLC–MS/MS) platform. Chromatographic separation was achieved on a Waters ACQUITY UPLC system coupled to a Xevo TQ-S triple quadrupole mass spectrometer (Waters Corp., Milford, MA). A Waters ACQUITY UPLC BEH C_18_ column (2.1 mm × 100 mm, 1.7 μm particle size) was used for metabolite separation. The mobile phase composition, gradient elution program, injection volume, column temperature, and mass spectrometer source parameters were set according to standard operating conditions of the platform. Mass spectrometric detection was conducted with an electrospray ionization source in multiple reaction monitoring mode to selectively detect and quantify targeted metabolite ion pairs.

Before UPLC–MS/MS analysis, internal standard compounds of known concentrations were added to each metabolite extract. Certain metabolites were derivatized where necessary to enhance detection sensitivity, according to their chemical properties. All samples were run in a randomized order to minimize batch effects. The analytical sequence included periodic injection of ­quality control samples, pooled reference samples, blank samples, and calibration standards to monitor instrument stability and to calibrate quantification throughout the run. Raw mass spectrometry data were processed using TMBQ software to quantify metabolite concentrations. Subsequent statistical analyses were performed using the iMAP metabolomics data analysis platform. Metabolite levels in LysoTag-positive versus LysoTag-negative samples were compared. After subtracting background signals from the negative controls, metabolites significantly enriched in the LysoTag-positive group were identified as lysosome-specific metabolites.

### Metabolomics analysis

In this study, the lysosome-specific metabolite concentrations were firstly log_10_ transformed, followed by PLS-DA based on the ropls package of R v4.5.1 (results were visualized by ggplot2). After assessing the characteristics of the data distributions by the Shapiro-Wilk test and Levene’s test, the results were selected for analysis of variance based on the test results by Student’s *t*-test (normal distribution with homogeneous variance), Welch’s *t*-test (normal distribution with heterogeneous variance), or the Wilcoxon rank-sum test (non-normal distribution). Candidate ­metabolites were screened with thresholds of |log_2_(FC)| > 1, *P *< 0.05, and VIP > 0.9; differential metabolite levels were revealed by volcano plots and heatmaps plotted by R v4.5.1. The differential metabolite functional enrichment analyses were performed in MetaboAnalyst 6.0. The metabolic pathway network relationship (Sankey diagram) and Spearman correlation cluster analysis visualization were performed through the online platforms (bioinformatics.com.cn/) and (omicstudio.cn), respectively.

### Transcriptomic analysis

The RNA-sequence data analyzed in this study are available in the National Center for Biotechnology Information’s Gene Expression Omnibus and can be accessed at ncbi.nlm.nih.gov/geo. Firstly, ENSEMBL IDs were extracted from the downloaded raw counts data, and then were converted to SYMBOL and ENTREZID via the org. Mm.eg.db annotation package in R v4.5.1. A conserved strategy was applied to the multi-mapping relationships (retaining the first valid annotation for each ENSEMBL ID). Subsequently, differential analyses were performed using the DESeq2 package, where low-expressed genes were filtered during the preprocessing stage (threshold set to count values > 10 in at least three samples) and differential significance was calculated by a negative binomial distribution model. Metabolic pathway genes, lysosomal genes, and autophagy-related genes were obtained from the KEGG database (kegg.jp/), as well as related publications [71, 72], respectively. Multivariate Venn diagrams (bioinformatics.com.cn/), binary plots (chiplot.online), and hierarchical clustering heatmaps integrating log_2_(FC) histograms (omicstudio.cn) were generated and visualized on the relevant online platforms. Finally, the key gene sets were imported into the STRING database to construct PPI networks, and the topological property calculation (node degree centrality) and modular visualization were performed using Cytoscape software, so as to mine the core regulatory genes.

### Statistical analysis

Data were analyzed using Prism (GraphPad Software) to gene­rate curves and bar charts. Statistical analysis was performed using *t*-tests or ANOVA (analysis of variance). One-way ANOVA was followed by pairwise *post-hoc* tests for each dependent variable. *P *< 0.05, indicated by ^*^, is considered statistically significant. *P *< 0.01, indicated by ^**^, is considered highly significant. *P *< 0.001, indicated by ^***^, is considered extremely significant. *P *> 0.05 is considered non-significant (ns).

## Supplementary Material

loag005_Supplementary_Data

## Data Availability

All study data are included in the article and/or supplementary information. Materials are available upon request.
