# Supplementary material for: Lysosomes contribute to the synthesis of tricarboxylic acid-related metabolites in the hippocampus
Source: Life Metab. 2026 Feb 19;5(3):loag005. doi: 10.1093/lifemeta/loag005 (PMC13134384; doi:10.1093/lifemeta/loag005)
Supplement: loag005_Supplementary_Data [file loag005_supplementary_data.zip › Supplementary_Information_-_LY_! - tu.docx]

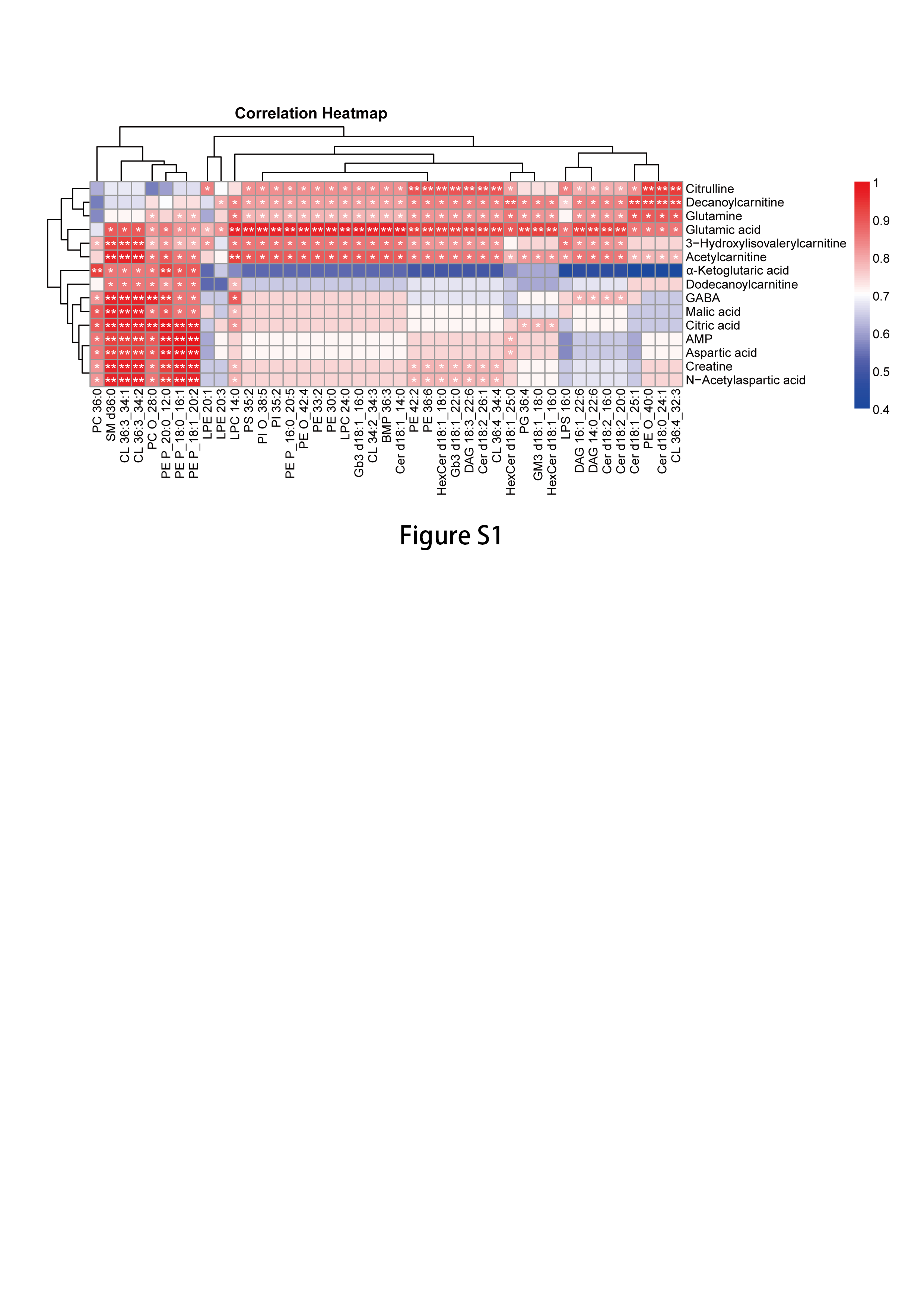


**Supplementary Figure S1** Spearman correlation cluster analysis of all significant metabolites.

**Supplementary Figure S2** CS is not expressed in lysosomes under physiological conditions. (a) The concentrations of citric acid in hippocampal lysosomes isolated from AL and EODF mice. Data are presented as mean ± s.e.m. *n* = 4 for AL group; *n* = 3 for EODF group. (b) Under HBSS treatment for 0 h or 6 h, with 3-MA or Mdivi-1 added as indicated, Lyso-IP isolates were subjected to metabolomic analysis to quantify citric acid concentrations (μmol/L). Each dot represents an independent experiment. (c) Citric acid levels were independently quantified using an enzyme-based assay kit and normalized to cell number (nmol/10^7^ cells). Each dot represents an independent experiment. (d) Immunoblot analysis of lysosomes isolated from HT22 cells using antibodies against the indicated proteins. (e−g) Representative images of the subcellular localization of CS in HT22 cells (e). White arrows indicate colocalization of the mitochondria with CS, whereas yellow arrows indicate CS delivery to the lysosomes via the autophagy pathway. Quantification of CS signals in LAMP1⁺ TOM20⁺ compartments representing mitophagy-associated pools, and in LAMP1⁺ TOM20^−^ lysosomes representing non-mitophagic pools (f and g). At least 15 cells were analyzed per condition. Scale bar, 1 μm. Endogenous proteins (blue) were detected by immunofluorescence. Lysosomes were labeled with LAMP1-GFP (green) and the mitochondria were labeled with TOM20-mScarlet3 (red). (h−k) Immunoblot analysis of lysosomes isolated from HT22 cells using antibodies against the indicated proteins. FH, IDH1/2, CS, and OGC levels were quantified and normalized to that of HBSS 0 h. HT22 cells were subjected to HBSS-induced starvation for 6 h, with or without Mdivi-1 treatment (10 μmol/L). Data are presented as mean ± s.e.m. Statistical analysis was performed using the two-tailed unpaired Student’s *t*-test or one-way/two-way ANOVA, as appropriate. ^*^*P* < 0.05; ^**^*P* < 0.01; ^***^*P* < 0.001; ^****^*P* < 0.0001.

**Supplementary Table S1** Information on antibodies used in this study.

| **Antibody**  **target** | **Company** | **Catalogue number** | **Dilution used in this study** |
| --- | --- | --- | --- |
| VDAC | ProteinTech | 10866-1-AP | WB 1:1000 |
| Cathepsin D | ProteinTech | 21327-1-AP | WB 1:5000 |
| calreticulin | ProteinTech | 10292-1-AP | WB 1:1000 |
| golgin 97 | ProteinTech | 12640-1-AP | WB 1:2000 |
| Catalase | ProteinTech | 21260-1-AP | WB 1:5000 |
| RPS6 | ProteinTech | 66886-1-Ig | WB 1:5000 |
| Histone H3 | ProteinTech | 17168-1-AP | WB 1:5000 |
| LAMP1 | abcam | ab24170 | WB 1:1000 |
| FH | CST | #4567 | WB 1:1000 IF 1:50 |
| IDH1 | CST | #3997 | WB 1:1000 IF 1:50 |
| CS | CST | #14309 | WB 1:1000 IF 1:50 |
| OGC | ProteinTech | 12253-1-AP | WB 1:1000 IF 1:50 |
