# Supplementary figures and images for: Lysosomes contribute to the synthesis of tricarboxylic acid-related metabolites in the hippocampus
Source: Life Metab. 2026 Feb 19;5(3):loag005. doi: 10.1093/lifemeta/loag005 (PMC13134384; doi:10.1093/lifemeta/loag005)

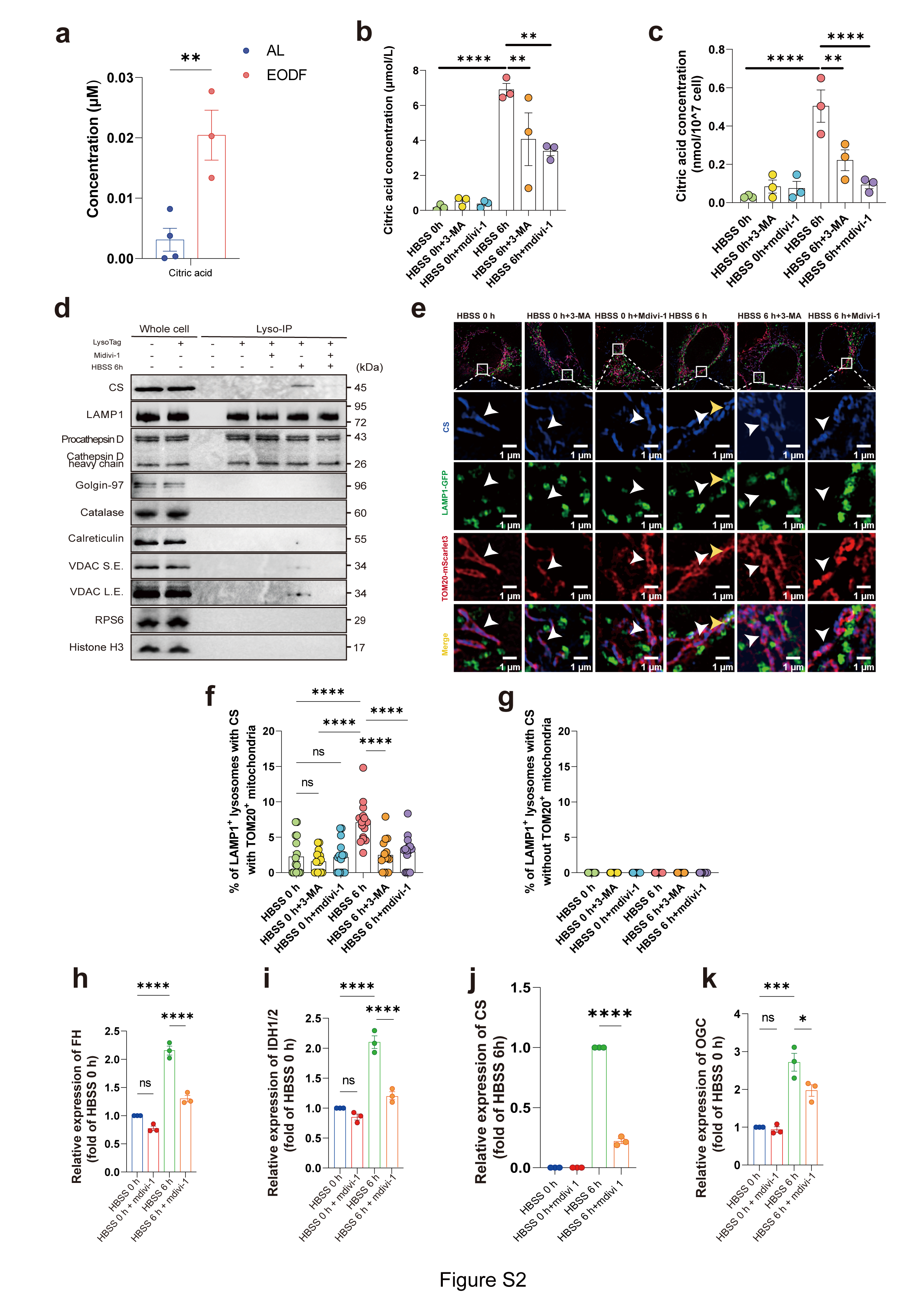

Supplement: loag005_Supplementary_Data [file loag005_supplementary_data.zip › figure s2.tif]

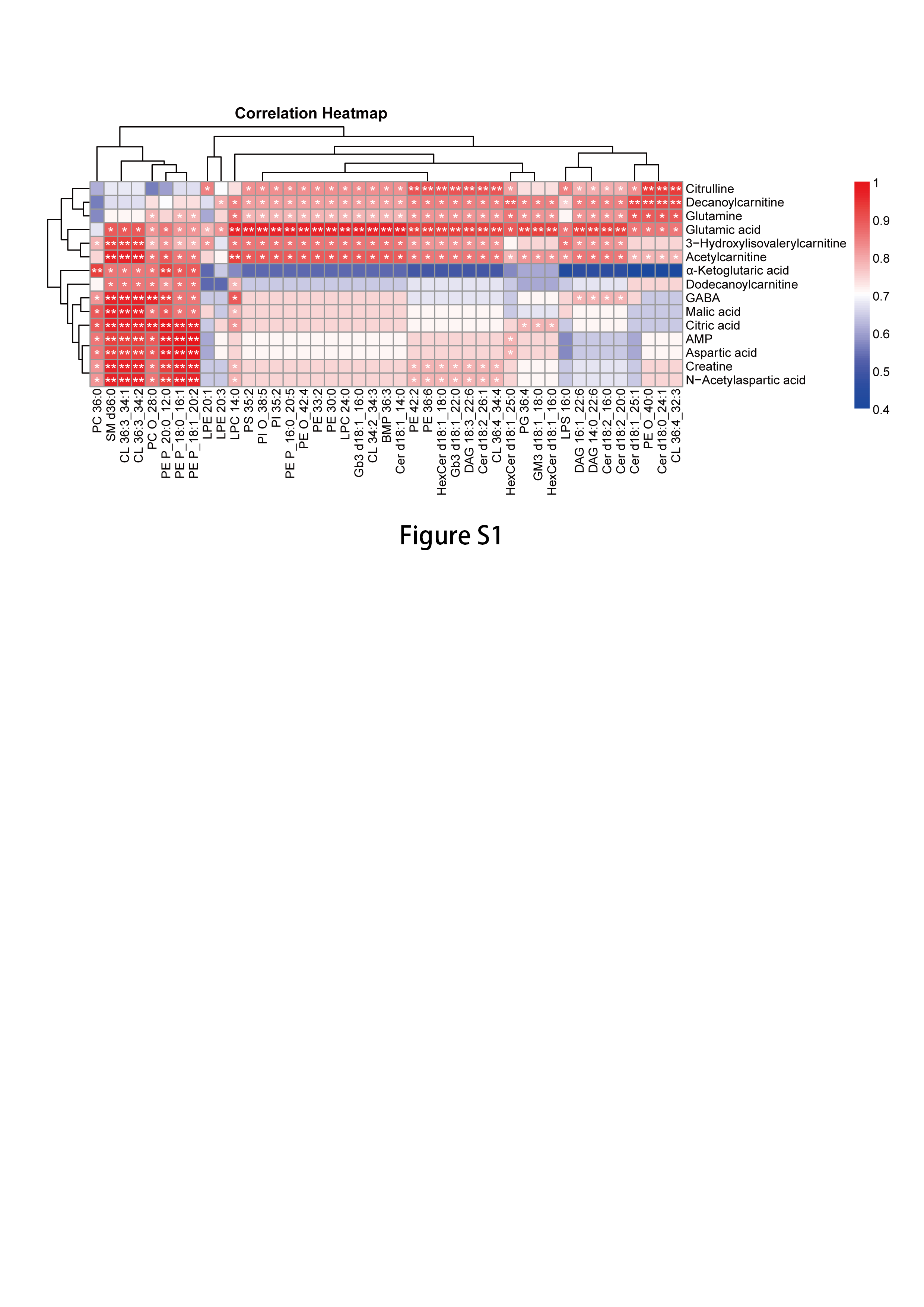

Supplement: loag005_Supplementary_Data [file loag005_supplementary_data.zip › Figure S1.tif]
